# Mumps Infection With Symptoms of Parotitis, Pancreatitis, and Orchitis Concurrently in an Adolescent Male

**DOI:** 10.7759/cureus.21963

**Published:** 2022-02-06

**Authors:** Tulika Garg, Monica Gupta, Samiksha Gupta, Narinder Kaur, Rayidi Rajesh

**Affiliations:** 1 Internal Medicine, Government Medical College and Hospital, Chandigarh, IND; 2 lnternal Medicine, Government Medical College and Hospital, Chandigarh, IND; 3 Radiodiagnosis, Government Medical College and Hospital, Chandigarh, IND

**Keywords:** effectiveness, efficacy, immunity, vaccine, mumps

## Abstract

Mumps is a highly contagious childhood infectious disease caused by the mumps virus. Clinical symptoms of mumps infection among vaccinated young adults are rarely seen. We present an unusual case of a vaccinated young male who presented with a clinical picture suggestive of mumps infection with symptoms of parotitis, pancreatitis, and orchitis. The waning of vaccine-induced immunity and low efficacy of the mumps component of the measles, mumps, and rubella (MMR) vaccine could be the reasons for the same. Our patient was managed with supportive measures for the complications and made an uneventful recovery. It has been postulated that antigenic differences between the vaccine and strain-causing illness may result in a deficient immune response conferred by the vaccine. This case highlights the concerns regarding the effectiveness of the live attenuated vaccine currently in use.

## Introduction

Mumps is an acute systemic viral illness caused by the ribonucleic acid (RNA) virus, Rubulavirus belonging to the family *Paramyxoviridae*. It mainly targets children in the age group of 2-9 years, who then usually acquire a natural active immunity [1]. Even though it is highly contagious, it can be prevented via vaccination. The clinical symptomatology of a mumps infection typically begins with prodromes of fever, fatigue, myalgia, and anorexia, followed by pain and swelling of one or more of the salivary glands, mainly the parotid glands. It can also result in serious complications, including orchitis, nephritis, pancreatitis, myocarditis, and neurological manifestations like meningitis encephalitis and deafness. In a few cases, long-term outcomes, such as hydrocephalus, seizures, and cranial nerve palsies, have also been reported [2,3]. Although it is a disease of the younger age group, we present a case of mumps in a vaccinated young adult.

## Case presentation

An 18-year-old male presented to our medical out-patient department with the complaints of low-grade intermittent fever, loss of appetite and multiple episodes of non-bilious vomiting of 10 days’ duration. This was followed by swelling below the left ear for seven days which was gradually progressive, painful, and ear pain aggravated while chewing. There was also a history of painful right scrotal swelling for the last three days. The patient did not have any history of abdominal pain, inhabiting closed environments, or international travel. His nasopharyngeal swab was negative for SARS-CoV-2 at the time of admission. He was vaccinated with MMR vaccine, first at the age of 15 months, and he received the second dose at the age of five years.

Physical examination revealed an average built male with an axillary temperature of 100°F, regular pulse rate of 105 beats/minute, blood pressure of 110/70mmHg, respiratory rate of 16/minute, and Glasgow coma scale of 15/15. On local examination, he had a diffuse, tender fluctuant, and erythematous swelling on palpation near the angle of the mandible on the left side. There was no visible purulent discharge from the Stenson’s duct. On systemic examination, the abdomen was tense with absent bowel sounds. The right testis on palpation was painful and swollen (approx. 5x3x3 cm). The rest of the systemic examination was grossly normal.

Initial laboratory investigations revealed a hemoglobin of 13 gm/dL (13-16 gm/dL), platelet count of 1.5 x105/L (1.5-4.0 x105/L), a total leucocyte count of 10x103cells/mm3 (4.0-10.0 x103cells/mm3), and erythrocyte sedimentation rate of 32 mm (0-20 mm) in the first hour. Serum amylase of 249 IU/L (23-125 IU/L), serum lipase 348 IU/L (13-60 IU/L), total bilirubin 0.55 mg/dL (0.2-1.0 mg/dL), and alkaline phosphatase 65 IU/L (40-130 IU/L). There was no hypercalcemia and hypertriglyceridemia: serum calcium 9.3 mg/dL (8.0-10.4 mg/dL) and triglycerides 72mg/dL (10-150 mg/dL). Laboratory confirmation of mumps virus RNA was done by oral swab through reverse transcriptase polymerase chain reaction (RT-PCR). Serologies for human immunodeficiency virus (HIV), Ebstein-Barr virus (EBV), cytomegalovirus (CMV), herpes simplex virus (HSV), influenza and parainfluenza were negative.

**Figure 1:**
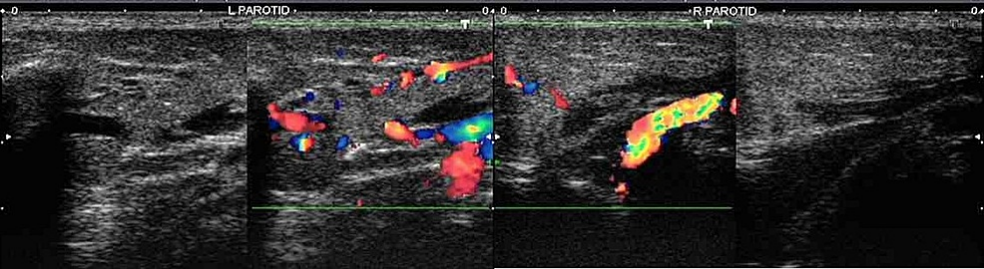
Ultrasound greyscale images showing diffuse hypoechogenicity and enlargement of the left and right parotid glands, and color Doppler images demonstrating increased vascularity (left>right) within parotid glands suggestive of acute parotitis

Ultrasound of the parotid glands (Figure 1) revealed enlargement with loss of normal echotexture and the presence of diffuse hypoechogenicity in bilateral parotid glands suggestive of parotitis.

**Figure 2:**
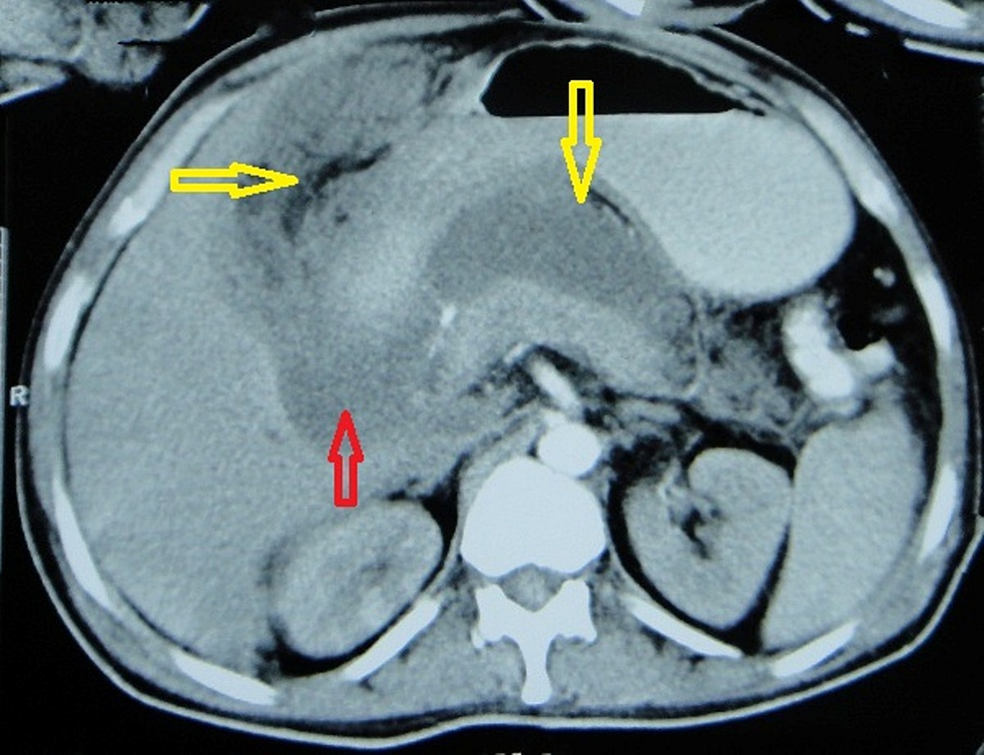
CECT abdomen axial scan at the level of the pancreas: There is mild focal hypodensity in the head and hypodensity in the tail of the pancreas suggestive of acute pancreatitis. There is fluid collection in the lesser sac (marked by vertical yellow arrow containing air specks on the non-dependent part [H.U. value around -302]), uncinate process (marked by red arrow), and right subhepatic space marked by the horizontal yellow arrow (containing fat and air specks within [H.U. value -50 to -290, respectively]).

The findings of pancreatitis were confirmed on computed tomography (CT) scan of the abdomen (Figure 2) done on day six of the illness. 

**Figure 3:**
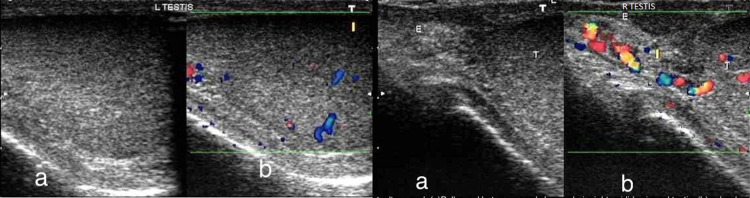
Ultrasound Left testis (a) Greyscale image showing hypoechoic echo pattern of left testis and (b) Color Doppler demonstrating increased vascularity. Ultrasound Right testis (a) Bulky and heterogeneously hypoechoic right testis and epididymis and (b) Color Doppler demonstrating increased vascularity in both structures.

Ultrasound of the left and right testis (Figure 3) showed swelling, heterogenous hypoechogenicity of the testis, and scrotal wall thickening. Color Doppler sonography was done simultaneously, and it revealed increased flow, hypervascularity, and hyperemia of the testis and epididymis on the right side. On the left side, only testicular hyperemia was observed. 

Based on the above findings, a diagnosis of mumps infection with parotitis, acute pancreatitis, and epididymo-orchitis was made.

The patient was hospitalised and isolated until the parotid swelling resolved. He was managed conservatively with antipyretics (acetaminophen) and analgesics (ibuprofen) along with a topical application of warm or cold packs for parotid discomfort. Acute pancreatitis was managed with adequate fluid resuscitation, while orchitis required bed rest, scrotal support, and cold packs with analgesics. The recovery was uneventful, and he was successfully discharged after five days of isolation.

## Discussion

Mumps is best known as a common childhood viral illness caused by the mumps virus. The mumps virus is a single-stranded, negative-sense, non-segmented RNA virus, Rubulavirus belonging to the family *Paramyxoviridae*. Mumps is principally a disease of children and young adults that is transmitted by direct contact with infectious droplet nuclei or through fomites containing infectious saliva [4]. The peak incidence is commonly seen in late winter or early spring, although the outbreaks can be seen any time of the year. Certain risk factors contributing to sporadic outbreaks include closed environments (e.g., schools, summer camps, college dormitories) as well as failure to recognize early symptoms of the disease [5]. The incubation period ranges from 12-25 days from exposure to onset of symptoms. The prodromal symptoms of low-grade fever, malaise, and headache are followed by parotid swelling, usually within 48 hours. Other symptoms include earache aggravated by chewing movements of the jaw or hearing loss as a result of the vestibular reaction. Although symptoms are similar, illnesses are usually more severe in young adults than in children [6]. Certain complications such as thyroiditis, pancreatitis, orchitis, oophoritis in post-pubertal females, myocardial involvement, arthritis, and neurological manifestation (aseptic meningitis, encephalitis, sensorineural hearing loss, transverse myelitis, facial palsy) have also been reported [1]. Since there is no specific antiviral treatment for mumps, management aims at supportive measures.

Mumps is a vaccine-preventable disease. Mumps vaccine had been introduced in 121 countries belonging to the World Health Organization (WHO) by the end of 2016 [7]. All available vaccines for mumps consist of live-attenuated mumps virus. Initially, one dose of MMR vaccine was recommended. Subsequently, in many countries, a second dose of the vaccine was added to the vaccination schedule later to optimize the immune response and to increase the proportion of individuals exhibiting antibodies. But it was revealed in a meta-analysis that the second application only restores immunity up to the level of the first vaccine [8]. In the United States of America (USA), the first MMR vaccine is given at the age of 12-15 months and the second at the age of 4-6 years. If the individual is lacking one or both vaccines, then the child should receive the MMR vaccine at 2-17 years of age. The vaccines are available in monovalent form or combination with other vaccines (used universally), such as the measles-mumps-rubella (MMR) combination.

The introduction of the vaccine was believed to have resulted in a decline in the incidence of measles, mumps, and rubella infections. But some recent reports have suggested the re-emergence of mumps infection worldwide in the vaccinated populations. The poor efficacy of the MMR vaccine has been proposed as a major contributor to the re-emergence of the outbreaks. It has been established that the median effectiveness of the mumps vaccine is 78% (49% to 92%) for one dose and 88% (66% to 95%) for two doses, which is lower than the effectiveness of its measles and rubella vaccine counterparts [9]. Possible reasons for the moderate effectiveness of the mumps vaccine include primary vaccine failure that is no seroconversion after vaccination as a result of the immature immune system, secondary failure (waning immunity), intense exposure to high virus inoculums, and a mismatch between vaccine genotype and circulating strain [10].

## Conclusions

This young adult presented with a clinical picture suggestive of parotitis, pancreatitis, orchitis, suggestive of florid mumps infection. It is known that the disease severity and incidence of complications of mumps are reduced significantly if an individual has received vaccination in the past. Although our patient had varied manifestations, the disease resolved spontaneously with supportive therapy. Considering this unusual constellation of complications of mumps in a previously vaccinated individual, the lifetime efficacy of the mumps component in the MMR vaccine needs more evaluation. 

## References

[1] Hviid A, Rubin S, Mühlemann K (2008). Mumps. Lancet.

[2] Betáková T, Svetlíková D, Gocník M (2013). Overview of measles and mumps vaccine: origin, present, and future of vaccine production. Acta Virol.

[3] Rubin S, Kennedy R, Poland G (2016). Emerging mumps infection. Pediatr Infect Dis J.

[4] Lane TM, Hines J (2006). The management of mumps orchitis. BJU Int.

[5] Bitsko RH, Cortese MM, Dayan GH, Rota PA, Lowe L, Iversen SC, Bellini WJ (2008). Detection of RNA of mumps virus during an outbreak in a population with a high level of measles, mumps, and rubella vaccine coverage. J Clin Microbiol.

[6] Gupta RK, Best J, MacMahon E (2005). Mumps and the UK epidemic 2005. BMJ.

[7] Burton A, Monasch R, Lautenbach B (2009). WHO and UNICEF estimates of national infant immunization coverage: methods and processes. Bull World Health Organ.

[8] Lewnard JA, Grad YH (2018). Vaccine waning and mumps re-emergence in the United States. Sci Transl Med.

[9] Demicheli V, Rivetti A, Debalini MG, Di Pietrantonj C (2012). Vaccines for measles, mumps and rubella in children. Cochr Data Syst Rev.

[10] Quinlisk MP (2010). Mumps control today. J Infect Dis.

